# Integrated Bioinformatic Analysis Reveals TXNRD1 as a Novel Biomarker and Potential Therapeutic Target in Idiopathic Pulmonary Arterial Hypertension

**DOI:** 10.3389/fmed.2022.894584

**Published:** 2022-05-12

**Authors:** Wenchao Lin, Yiyang Tang, Mengqiu Zhang, Benhui Liang, Meijuan Wang, Lihuang Zha, Zaixin Yu

**Affiliations:** Department of Cardiology, Xiangya Hospital, Central South University, Changsha, China

**Keywords:** bioinformatic analysis, TXNRD1, pulmonary hypertension, biomarker, RRA, GEO

## Abstract

Idiopathic pulmonary arterial hypertension (IPAH) is a life-threatening cardiopulmonary disease lacking specific diagnostic markers and targeted therapy, and its mechanism of development remains to be elucidated. The present study aimed to explore novel diagnostic biomarkers and therapeutic targets in IPAH by integrated bioinformatics analysis. Four eligible datasets (GSE117261, GSE15197, GSE53408, GSE48149) was firstly downloaded from GEO database and subsequently integrated by Robust rank aggregation (RRA) method to screen robust differentially expressed genes (DEGs). Then functional annotation of robust DEGs was performed by GO and KEGG enrichment analysis. The protein-protein interaction (PPI) network was constructed followed by using MCODE and CytoHubba plug-in to identify hub genes. Finally, 10 hub genes were screened including ENO1, TALDO1, TXNRD1, SHMT2, IDH1, TKT, PGD, CXCL10, CXCL9, and CCL5. The GSE113439 dataset was used as a validation cohort to appraise these hub genes and TXNRD1 was selected for verification at the protein level. The experiment results confirmed that serum TXNRD1 concentration was lower in IPAH patients and the level of TXNRD1 had great predictive efficiency (AUC:0.795) as well as presents negative correlation with mean pulmonary arterial pressure (mPAP) and pulmonary vascular resistance (PVR). Consistently, the expression of TXNRD1 was proved to be inhibited in animal and cellular model of PAH. In addition, GSEA analysis was performed to explore the functions of TXNRD1 and the results revealed that TXNRD1 was closely correlated with mTOR signaling pathway, MYC targets, and unfolded protein response. Finally, knockdown of TXNRD1 was shown to exacerbate proliferative disorder, migration and apoptosis resistance in PASMCs. In conclusion, our study demonstrates that TXNRD1 is a promising candidate biomarker for diagnosis of IPAH and plays an important role in PAH pathogenesis, although further research is necessary.

## Introduction

Pulmonary arterial hypertension (PAH) is a progressive cardiovascular disease characterized by vasodilation dysfunction and irreversible vascular remodeling, eventually leading to pulmonary pressure elevation and right heart failure (1). According to WHO classification, PAH is divided into five groups and idiopathic pulmonary arterial hypertension (IPAH) is the most common subtype of PAH occurred in the absence of etiology (2). Although considerable target therapy for PAH have been developed to well relieve symptoms, this devastating disease still carries a poor prognosis with a 5-year survival of 61.2% in newly diagnosed patients (3). In the early stage of PAH, pulmonary vasculature presents sustained constriction which can be reversed by anti-vasoconstrictor drugs. As the disease progresses, vascular remodeling including distal artery hypertrophy, inflammation, fibrosis, and neovascularization, predominately accelerate the deterioration of the disease (4). Current therapies mainly focus on improving pulmonary vasodilation rather than targeting proliferative vascular remodeling and that is the reason why target therapy gradually lose efficacy in the advanced stage of the disease (5). Therefore, there is urgent need to explore novel therapeutic target to directly address the pathological remodeling that underpins the disease.

In the past decades, microarray and RNA sequencing technology have been widely used for gene expression analysis in lung tissue or cell from PAH patients or experimental animal, which has already identified multiple therapeutic genes target and yielded some achievements in pathogenesis knowledge (6–8). However, currently most PAH lung tissue gene expression profiles have not yet been uploaded, and analysis among the uploaded datasets are very inconsistent due to the limited sample size. Therefore, it is very necessary to integrate the existing PAH datasets. Robust rank aggregation (RRA) method is a dataset integration method based on gene ranking list comparison. It hypothesizes that each gene is randomly ordered, if a gene is upregulated or downregulated in all gene lists, then the gene is considered robust (9). Up to now, there have been several studies that integrated PAH datasets adopting batch normalization method for consolidation while there are rare studies using RRA method for data integration (10, 11), which facilitated this study.

In this study, we performed an integrated bioinformatics analysis to identify robust differentially expressed genes (DEGs) in IPAH. The gene expression profiles of GSE117261, GSE53408, GSE48149, and GSE15197 were downloaded from GEO database. After screening DEGs in each dataset, the RRA method was applied to identify 169 robust DEGs and enrichment analysis was conducted. Then, we uploaded robust DEGs to the STRING database to construct global protein-protein interaction network. The module analysis was performed by the MCODE plug-in of Cytoscape based on the entire network and hub genes identification was carried out by the plug-in Cytohubba. Through verification of independent dataset and experimental validation, TXNRD1 was finally identified as a candidate molecular biomarker and potential therapeutic target in IPAH.

## Materials and Methods

### Gene Expression Omnibus Datasets Selection

We screened the Gene Expression Omnibus (GEO) database^1^ according to the following inclusion criteria: (1) Samples from human lung tissue; (2) Containing at least five IPAH cases and at least five controls; (3) Raw data or gene expression profiling were available for downloading in GEO. Finally, a total of 5 datasets were obtained: GSE117261, GSE53408, GSE48149, and GSE15197 were used for RRA analysis, and GSE113439 was used as the validation dataset. The detailed information of datasets was listed in Table 1.

**TABLE 1:** Datasets detailed information.

References	Sample	GEO	Platform	IPAH	Control
Stearman et al.	Lung tissue	GSE117261	GPL6244	32	25
Hsu et al.	Lung tissue	GSE48149	GPL16221	8	9
Rajkumar et al.	Lung tissue	GSE15197	GPL6480	13	18
Zhao et al.	Lung tissue	GSE53408	GPL6244	12	11
Mura M. et al.	Lung tissue	GSE113439	GPL6244	6	11

### Identification of Robust Differentially Expressed Genes Among Each Gene Expression Omnibus Dataset

For each dataset, we firstly downloaded the gene expression matrix and annotation document from GEO database, then the microarray probes were mapped to corresponding gene symbol and the not available gene symbols were removed from the expression matrix. If multiple probes annotated with the same symbol, the mean value was adopted. To determine DEGs between IPAH and control group, we utilized limma R package to analyze with the cut-off criteria of | log2 fold-change (FC)| > 0.5 and *P*-value < 0.05. Identified DEGs from each dataset were integrated using the Robust Rank Aggregation (RRA) method to minimize the inconsistency. RRA analysis was performed with an R package “Robust Rank Aggreg.” Genes with *P*-value < 0.05 were regarded as robust DEGs.

### Gene Ontology and Kyoto Encyclopedia of Genes and Genomes Pathway Enrichment Analysis

In order to excavate the function of robust DEGs, we performed Gene Ontology (GO) enrichment analysis and Kyoto Encyclopedia of Genes and Genomes (KEGG) pathway analysis *via* R package “clusterprofiler” (12). Terms with *P* < 0.05 was considered to be significant enrichment.

### Protein-Protein Interaction Network Analysis

The organization of protein-protein interaction (PPI) network was managed by the Search Tool for the Retrieval of Interacting Genes (STRING^2^). Interaction with combined score ≥ 0.4 was set as cut-off point. To visualize the global PPI network, Cytoscape (3.8.0) software was applied. The MCODE plug-in of Cytoscape software was used to identify significant modules with default parameters (degree of cut off = 2, node score cutoff = 0.2, k-core = 2, and max depth = 100). To seek for important hub genes among robust DEGs, the Cytohubba plug-in of Cytoscape software was applied, which provided top 10 hub genes by combing different algorithm.

### Clinical Blood Sampling and Enzyme-Linked Immunosorbent Assay

To test whether TXNRD1 was reduced in the blood of IPAH patients, 9 diagnosed IPAH patients were recruited from the Department of Cardiology of the Xiangya Hospital, and 13 healthy controls with matched age and gender were recruited during physical check-up. The patient data and samples collection were approved by the Medical Ethics Committee of the Xiangya Hospital of Central South University and informed consent was obtained from all subjects or their legal guardians. All methods were carried out in accordance with the approved guidelines. Clinical features include age, sex, BMI, 6-min walk distance (6MWD), NT-proBNP, mPAP, and PVR. Blood samples were collected through vein puncture and subsequently processed to serum as soon as possible. TXNRD1 levels were determined using the human TrxR enzyme immunoassay kit (CSB-E09731h, CUSABIO, Wuhan, China) according to the manufacturer’s instructions. Receiver operating characteristic (ROC) curve analysis was completed by the Hipplot online tool^3^ to determine the sensitivity and specificity of TXNRD1.

### Animal Experiment

This study was approved by the Institutional Animal Care and Use Committee of Central South University. Fourteen 180–200 g male SD rats were raised under standard laboratory conditions and followed the National Institutes of Health guide for the care and use of rat. One week later, rats were randomly divided into control group and MCT group (*n* = 7) which received intraperitoneal injection with saline and MCT (60 mg/kg, Sigma-Aldrich, St Louis, MO, United States) respectively. After 21 days, the rats were anesthetized and the right ventricle systolic pressure (RVSP) was measured through right heart catheterization. Then, the rats were sacrificed, and lung and heart tissues were collected for histological analysis and western blotting. The RV hypertrophy index was calculated as the weight ratio of RV to (LV + ventricular septum).

### Cell Culture Experiments

Primary rat PASMCs were isolated and cultured following previously described protocol (13). Briefly, rats were sacrificed under anesthetized condition and lung tissue were quickly separated. Then the distal pulmonary arteries were peeled off and connective tissue was removed in PBS. Pulmonary artery was then cut into small pieces and incubated in HBSS containing 1 mg/ml collagenase I (Sigma-Aldrich, St Louis, MO, United States) at 37°C for 20 min under shaking until the tissue block is digested. Tissue lysate was then filtered through 0.45 μm cell strainer and centrifuged to collect cells which were cultivated in DMEM/F12 containing 20% fetal bovine serum (FBS) till reaching confluence. Cell purity was confirmed by immunofluorescence with smooth muscle cell actin (1:400, 67735-1-Ig, ProteinTech). PASMCs of passages 3–5 at 70–80% confluence were used for experiments. As for stimulation of PASMC proliferation, cells were starved in 0.5% FBS supplemented medium for 24 h and then treated with 20 ng/ml PDGF-BB (Peprotech, Rocky Hill, NJ, United States) for 24 h. For gene knockdown in PASMCs, cells were transfected with control siRNA or si-TXNRD1 in antibiotic-free medium for 48 h following the manufacturer’s instructions. The siRNA was synthesized by Ribobio (China) and the sequence was as follows: si-TXNRD1, 5′-GGAAGAGATTCTTG TACAA-3′.

### Western Blot

For the analysis of total proteins, lung tissue or PASMC were lysed in RIPA containing protease inhibitor. Following this, the lysates were centrifuged at 12,000*g* for 10 min, and the supernatant was collected. The BCA Protein Assay Kit (Beyotime) was used to determine protein concentrations. Total lysates were loaded on 10% SDS-PAGE to separate the proteins electrophoretically and the proteins were then transferred to a PVDF membrane. Blocked membranes in 5% bovine serum albumin were probed using the following antibodies: TXNRD1 (1:1,000, sc-28321, Santa Cruz Biotech), PCNA (1:2000, 10205-2-AP, Proteintech), Bcl-2 (1:1000, 60178-1-Ig, Proteintech), BAX (1:5000, 60267-1-Ig, Proteintech), cleaced-PARP (1:1000, 94885, CST), β-actin (1:2000, 20536-1-AP, Proteintech). Reactive bands were visualized with the chemiluminescent protocol, recorded with the ChemiDoc MP Imaging System (Bio-Rad).

### RNA Extraction and Real-Time qPCR

In order to assess the knockdown efficiency of TXNRD1, total RNA was extracted from PASMCs using RNA Extraction Kit (#6834, OMEGA biotek) according to the manufacturer’s instructions. First-strand cDNA was reverse transcribed from total RNA using the First-Strand cDNA Synthesis Kit (GeneCopeia). mRNA level of TXNRD1 was quantified by Real-time PCR using SYBR Green qPCR Mix Kit (GeneCopeia) on QuantStudio 5 (Thermo) with following primer: 5′-GGTGAAAGGCCGCGCTA-3′ (forward), 5′ATAGGACGCGCCAACCACTA-3′ (reverse). Data were analyzed using the 2-ΔΔCT method with GAPDH as an internal control.

### Gene Set Enrichment Analysis

Gene set enrichment analysis was conducted using “clusterprofiler” to explore the potential signaling pathways related to TXNRD1 in IPAH. The MSigDB gene set, “h.all.v7.4.entrez.gmt,” was defined as reference. Terms with *P* < 0.05 and FDR < 0.25 were defined as significant.

### Cell Proliferation and Migration Assays

EdU assay kit (Ribobio) was used to measure the PASMC proliferation according to manufacturer’s instruction (Ribobio, China). Briefly, PASMCs were cultured in 24-well culture plates and were subcultured with EdU labeling solution for 2 h after 48 h transfection with or without PDGF-BB stimulation. Then the cells were fixed by paraformaldehyde and incubated with EdU detection solution for 1 h. The stained cells were examined using a fluorescent-inverted microscope. Cell proliferation rate was calculated as the number of EdU-stained cells/the number of Hoechst–stained cells. Cell migration was determined by the wound-healing assay. In brief, cell medium was switched to starvation medium with or without PDGF-BB treatment after transfection, then a line scratch was drawn in the cell layer and photographed immediately as initial image (0 h). The second image (24 h) of the same line scratch was taken after 24 h of culturing, and cell migration was determined by measuring the decreased width of corresponding scratch from 0 to 24 h.

### Statistical Analysis

Statistical analysis was conducted using R software (Version 3.5.3) and GraphPad Prism 8. Student *t*-test and one-way ANOVA were used to compare two and multiple groups. Pearson’s correlation test and linear regression analysis were applied to specify the relationships between TXNRD1 levels and clinical parameters. Data was presented as mean ± SEM and *P* < 0.05 was considered statistically significant.

### Data Availability

The authors declare that all data supporting the findings of this study are available in Zenodo (DOI: 10.5281/zenodo.5840973). If any other requirements were requested, please contact the author at convenience.

## Results

### Identification of Differentially Expressed Genes Among Each Selected Dataset

**FIGURE 1:**
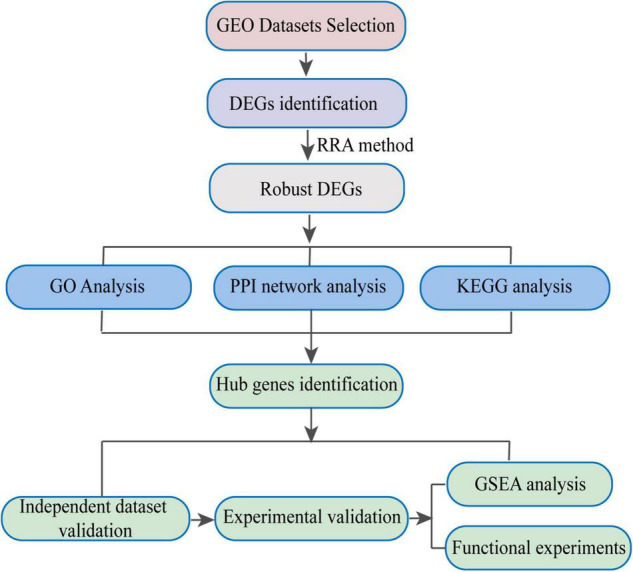
Workflow of the present study. GEO, Gene Expression Omnibus; DEGs, Differentially Expressed Genes; RRA, Robust Rank Aggregation; PPI, Protein-Protein Interaction; GSEA, Gene Set Enrichment Analysis.

**FIGURE 2:**
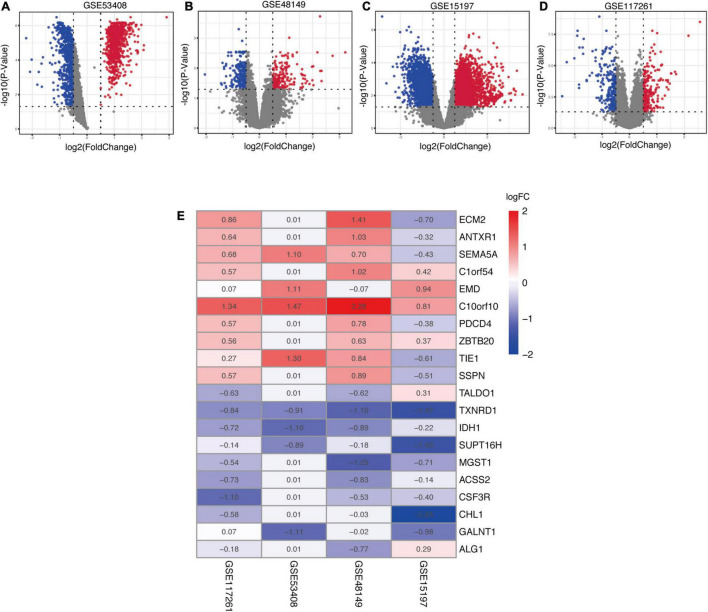
Identification of robust DEGs among each GEO dataset. **(A–D)** The volcano plots of the distribution of DEGs in each dataset. **(E)** The heatmap of robust DEGs in the RRA analysis. The values in a cell represent |log2 fold-change (FC)| of each gene in a dataset. DEG, differentially expressed gene; GEO, Gene Expression Omnibus; RRA, robust rank aggregation.

In the present study, robust DEGs and hub genes were determined based on various GEO datasets by integrated bioinformatics analysis (Figure 1). After filtering the GEO datasets based on the given eligibility criteria, four PAH-associated datasets were included, and their detailed information was listed in Table 1. There was a total of 128 profiles including 65 IPAH patients and 63 controls selected for data processing. After the normalization and annotation of expression matrix, we used the “limma” package to screen DEGs among each dataset. Overall, 501 DEGs including 235 upregulated and 266 downregulated genes were selected in GSE117261 dataset (Supplementary Table 1). There were 963 DEGs in the GSE48149 dataset, including 552 upregulated and 411 downregulated genes (Supplementary Table 2). Additionally, 1562 DEGs were screened from the GSE53408 dataset including 858 upregulated and 704 downregulated genes (Supplementary Table 3). A total of 5971 DEGs were found in GSE15197 dataset including 3214 upregulated and 2847 downregulated genes (Supplementary Table 4). The volcano plots of DEGs among each dataset were shown in Figures 2A–D.

### Selection of Robust Differentially Expressed Genes by Robust Rank Aggregation Method

In order to explore DEGs with similar expression pattern in these datasets, we applied RRA method to decrease inconsistency of DEGs screened from each dataset. Finally, a total of 169 significantly robust DEGs were determined including 98 upregulated and 71 downregulated genes (Supplementary Table 5). The top 10 upregulated and downregulated genes were illustrated by a heatmap (Figure 2E), and the heatmap of 169 robust DEGs was shown in Supplementary Figure 1. Among the robust upregulated genes, ECM2 was ranked as the first one (*p* = 5.91E-05), followed by ANTXR1 (*p* = 4.33E-04) and SEMA5A (*p* = 1.05E-03). Meanwhile, TALDO1 (*p* = 5.99E-05), TXNRD1 (*p* = 1.66E-04), and IDH1 (*p* = 5.46E-04) were ranked as the top three robust downregulated genes.

### Enrichment Analysis of Robust Differentially Expressed Genes

**FIGURE 3:**
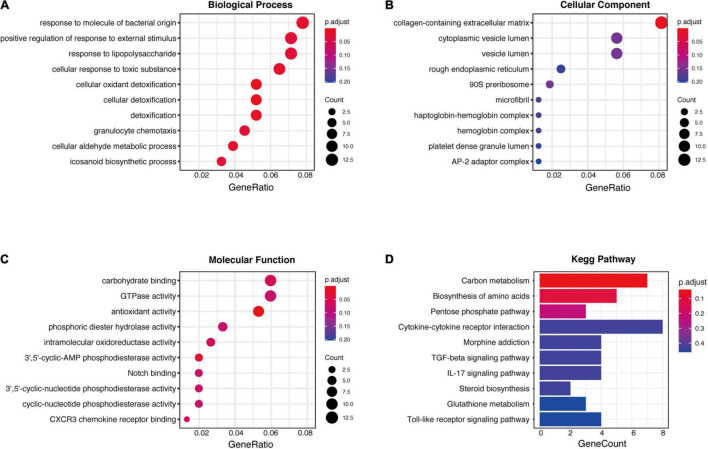
GO and KEGG enrichment analysis of robust DEGs. **(A)** Biological process GO terms for robust DEGs. **(B)** Cellular component GO terms for robust DEGs. **(C)** Molecular function GO terms for robust DEGs. **(D)** KEGG analysis for robust DEGs. DEG, differentially expressed gene; GO, Gene Ontology; KEGG, Kyoto Encyclopedia of Genes and Genomes.

To explore the biological role of selected robust DEGs in IPAH, we performed GO annotation and KEGG pathway enrichment analysis. Several biological processes in GO terms were enriched, such as response to molecule of bacterial origin, response to lipopolysaccharide and cellular oxidant detoxification (Figure 3A). Collagen-containing extracellular matrix was the most enriched cellular component in GO terms (Figure 3B). In terms of molecular function, antioxidant activity and 3′,5′-cyclic-AMP phosphodiesterase activity were identified as the significant enriched GO terms (Figure 3C). In the KEGG analysis, the robust DEGs were mostly associated with carbon metabolism (Figure 3D). The detailed results of enrichment analysis were shown in Table 2. The above results indicated that the robust DEGs were significantly enriched in PAH-related biological processes.

**TABLE 2:** GO and KEGG enrichment terms.

Term	Description	Gene Count	Adjusted *P*-value
GO:0098869	Cellular oxidant detoxification	8	0.006
GO:1990748	Cellular detoxification	8	0.0062
GO:0098754	Detoxification	8	0.0133
GO:0002237	Response to molecule of bacterial origin	12	0.0202
GO:0006081	Cellular aldehyde metabolic process	6	0.0178
GO:0097237	Cellular response to toxic substance	10	0.0178
GO:0071621	Granulocyte chemotaxis	7	0.0254
GO:0032103	Positive regulation of response to external stimulus	11	0.0254
GO:0046456	Icosanoid biosynthetic process	5	0.0254
GO:0032496	Response to lipopolysaccharide	11	0.0254
GO:0001569	Branching involved in blood vessel morphogenesis	4	0.0299
GO:0006739	NADP metabolic process	4	0.0299
GO:0071216	Cellular response to biotic stimulus	9	0.0316
GO:0097530	Granulocyte migration	7	0.0327
GO:0030593	Neutrophil chemotaxis	6	0.0348
GO:0006098	Pentose-phosphate shunt	3	0.0348
GO:0019321	Pentose metabolic process	3	0.0348
GO:0046184	Aldehyde biosynthetic process	3	0.0348
GO:0062023	Collagen-containing extracellular matrix	13	0.0080
GO:0016209	Antioxidant activity	8	0.0002
GO:0004115	3′,5′-cyclic-AMP phosphodiesterase activity	3	0.0236
hsa01200	Carbon metabolism	7	0.0403

### Protein-Protein Interaction Network Establishment and Hub Genes Identification

**FIGURE 4:**
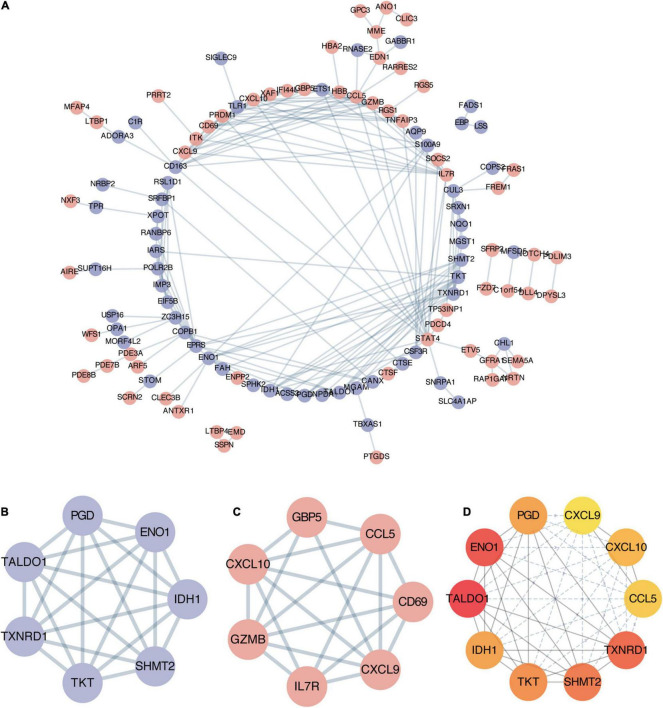
Establishment of protein-protein interaction (PPI) network and hub genes identification. **(A)** Entire PPI network of robust DEGs. The red nodes represent upregulated DEGs and the purple nodes represent downregulated DEGs. **(B)** The significant module 1 identified from PPI network *via* MCODE (score = 7). **(C)** The significant module 2 identified from PPI network *via* MCODE (score = 6.3). **(D)** The top 10 hub genes with highest degrees identified by cytoHubba analysis, the shade of red color represents the degree of importance.

To explore potential connection and identify hub genes among 169 robust DEGs, the STRING database was used to create global PPI network which was visualized by Cytoscape software (Figure 4A). Then MCODE plug-in was applied to find key modules (Figures 4B,C and Table 3). The genes in module 1 contains PGD, ENO1, TALDO1, IDH1, TXNRD1, TKT, SHMT2, which are associated with metabolic reprogramming and NADP metabolic process. The genes in module 2 correlates with cytokine-cytokine receptor interaction, including GBP5, CCL5, CD69, CXCL9, IL7R, GZMB, CXCL10. In addition, we utilized cytohubba plug-in to further determine the top 10 hub genes of DEGs including ENO1, TALDO1, TXNRD1, SHMT2, IDH1, TKT, PGD, CXCL10, CXCL9, and CCL5 (Figure 4D).

**TABLE 3:** MCODE module.

	Genes	Description	log10 (*P*-value)
Module 1	PGD, ENO1, TALDO1, IDH1, TXNRD1, TKT, SHMT2	Carbon metabolism	−13.57
		Metabolic reprogramming	−12.81
		Biosynthesis of amino acids	−11.61
		NADP metabolic process	−10.16
Module 2	GBP5, CCL5, CD69, CXCL9, IL7R, GZMB, CXCL10	Cytokine-cytokine receptor interaction	−6.55

### Validation of TXNRD1 in Clinical Samples

**FIGURE 5:**
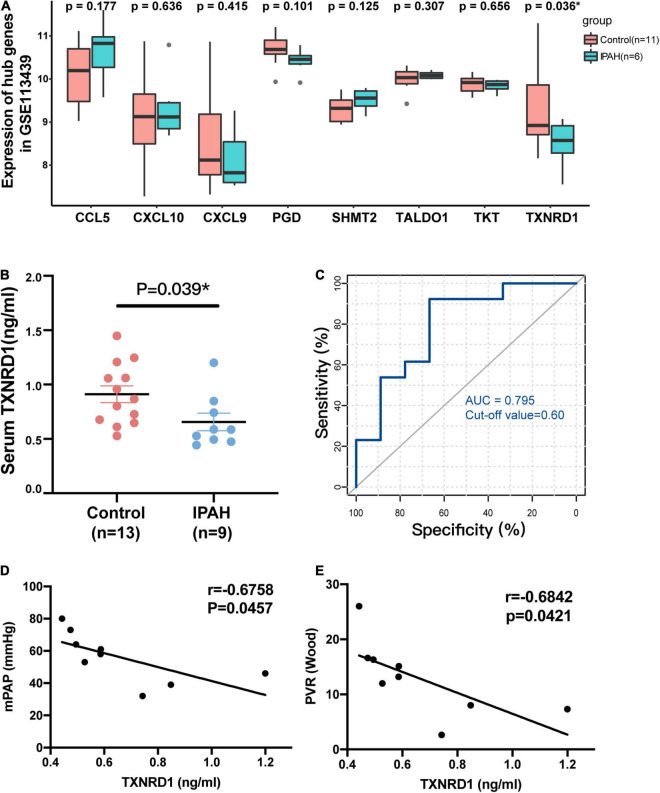
Validation of TXNRD1 in clinical samples. **(A)** Gene expression of hub genes in GSE113439. **(B)** Serum TXNRD1 was decreased in IPAH patients. **(C)** The ability of serum TXNRD1 levels to diagnose IPAH was assessed using ROC curve analysis. **(D,E)** The relationship between serum TXNRD1 and mPAP as well as PVR analyzed by Spearman’s correlation analysis.

Based on verification analysis in GSE113439, we found that TXNRD1 showed consistently downregulated in IPAH group while other hub genes showed no difference (Figure 5A). Therefore, we selected TXNRD1 for further experimental validation. Firstly, we collected the blood samples from 9 IPAH patients and 13 healthy controls to detect the serum TXNRD1 concentration. The clinical baseline characteristics of study subjects were shown in Table 4. As a result, we found that serum TXNRD1 concentration was lower in IPAH patients compared with healthy controls (Figure 5B). The ability of TXNRD1 levels to diagnose IPAH was assessed using ROC curve analysis which showed that the sensitivity and specificity were 92.3 and 66.7%, respectively, at the optimal expression cutoff value of 0.60, and AUC value of 0.795 exhibited great predictive efficiency of TXNRD1 as diagnosis biomarker (Figure 5C). We subsequently analyzed the relationship between serum TXNRD1 levels and clinical characteristics of IPAH patients, and the results revealed that TXNRD1 levels were negatively correlated with mPAP and PVR (Figures 5D,E) but had no significant correlation with 6MWD and NT-proBNP (Supplementary Figure 2).

**TABLE 4:** Clinical characteristics of the study population.

Characteristics	IPAH (*n* = 9)	Control (*n* = 13)	*P*-value
Age (years)	38.5 ± 3.6	34.4 ± 0.7	0.201
BMI (kg/m^2^)	20.6 ± 0.8	19.6 ± 0.3	0.236
NT-proBNP (pg/ml)	2414 ± 857		
6MMW (m)	409.5 ± 16.8		
mPAP (mmHg)	56.22 ± 5.18		
PVR (wood)	13.01 ± 2.25		

### *In vitro* and *in vivo* Experimental Validation and Gene Set Enrichment Analysis Analysis of TXNRD1

**FIGURE 6:**
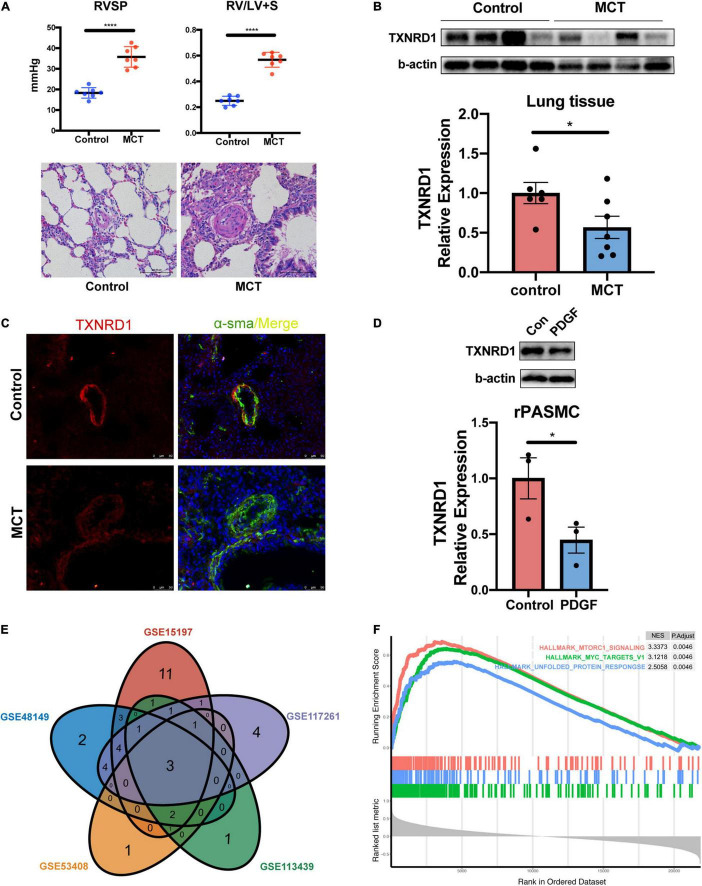
*In vitro* and *in vivo* experimental validation and GSEA analysis of TXNRD1. **(A)** The hemodynamic data and HE staining of lung tissue in animal experiments. **(B)** Representative Western blots and quantification of TXNRD1 and β-actin in the lungs of monocrotaline treated rats and controls (*n* = 7 each, *P < 0.05), data represent the mean ± SEM and Student *t*-test was used to compare two groups. **(C)** Immunofluorescence images of lung distal PA from MCT rats and controls. **(D)** Representative Western blots and quantification of TXNRD1 levels in rat PASMCs under PDGF-BB stimulation (30 ng/ml) normalized to a β-actin internal control (*n* = 3 each,*P < 0.05). **(E)** Venn diagram of GSEA terms among five datasets based on correlation analysis of TXNRD1. **(F)** The overlapping three GSEA terms correlated with TXNRD1. ****P < 0.0001.

Besides blood samples, TXNRD1 expression was also verified by Western blotting using lung homogenate of monocrotaline (MCT) treated rat which was a well-recognized animal model of PAH. As shown in Figure 6A, MCT-treated rats exhibited markedly increased RVSP and RV/(LV + S) as well as pulmonary vascular remodeling. Lung homogenates from MCT rats were measured by Western blot analysis and the results indicated TXNRD1 was significantly downregulated compared with controls (Figure 6B). The result was also confirmed by immunofluorescence staining in which TXNRD1 was predominantly expressed in the medial layer of vasculature (Figure 6C). Since excessive proliferation of pulmonary artery smooth muscle cell (PASMC) is a pivotal pathophysiological process of PAH, we isolated the rat PASMCs which were confirmed by immunofluorescence with alpha-sma (see Supplementary Figure 3) and stimulated it with PDGF-BB (platelet-derived growth factor-BB), a cytokine which has been proposed to be a key mediator of PASMC proliferation in the progression of PAH. Following PDGF-BB stimulation, we observed that TXNRD1 expression was suppressed, which was consistent with our previous results (Figure 6D). To decipher the molecular mechanisms leading to deregulation of TXNRD1 in PAH, we analyzed the correlation between TXNRD1 and other genes in multiple datasets and performed GSEA enrichment analysis. As shown in Figures 6E,F, there were three GSEA terms that were collectively enriched among different datasets, namely HALLMARK_MTORC1_SIGNALING, HALLMARK_MYC_TARGETS_V1, and HALLMARK_ UNFOLDED_PROTEIN_RESPONSE. The entire list of GSEA terms of each datasets could be found as Supplementary Tables 6–10.

### Knockdown of TXNRD1 Promotes Proliferation, Migration, and Apoptosis Resistance in Pulmonary Artery Smooth Muscle Cell

**FIGURE 7:**
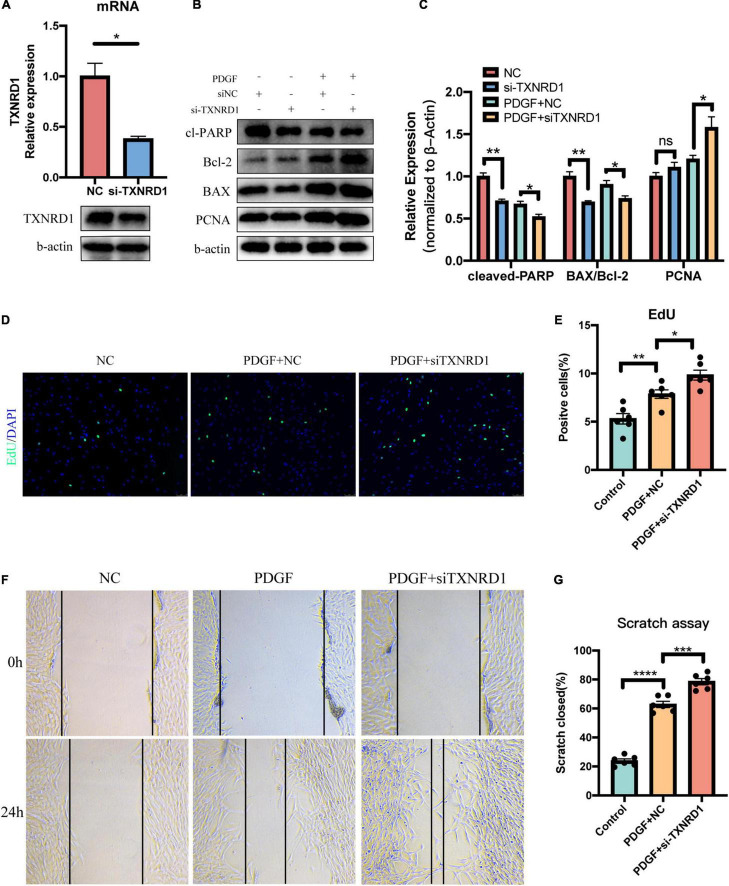
Silencing of TXNRD1 exacerbated PDGF-induced PASMC malignant phenotype. **(A)** Knockdown efficiency of TXNRD1 was verified by qPCR and Western Blot. **(B)** PASMCs were transfected with siRNA for 48 h before treatment with PDGF-BB (20 ng/ml) for another 24 h, and western blotting was used to detect proliferative and apoptotic markers in cell lysates. **(C)** Normalized quantification of cleaved-PARP, BAX/Bcl-2 and PCNA expression (*n* = 3 each, *P < 0.05). **(D)** Cell proliferative ability was determined by EdU assay in siTXNRD1-PASMC under PDGF stimulation. **(E)** Calculation of EdU-stained cells rate (*n* = 6 each, *P < 0.05). **(F)** Cell migration was measured by scratch assay in siTXNRD1-PASMC under PDGF stimulation. **(G)** Calculation of scratch closed percentage (*n* = 6 each, *P < 0.05). Data represent the mean ± SEM. Student *t*-test and one-way ANOVA were used to compare two and multiple groups. **P < 0.01, ***P < 0.001,****P < 0.0001.

In order to further explore the functions of TXNRD1 in PASMC, we knocked down the TXNRD1 in transcriptional level to evaluate if silencing TXNRD1 exacerbated PASMC malignant phenotype, including uncontrolled proliferation, migration and apoptosis resistance. We firstly silenced TXNRD1 in PASMC with siRNA that targets TXNRD1 (Figure 7A) and exposed them to normal condition or PDGF stimulation for 24 h. As shown in Figures 7B–E, knockdown of TXNRD1 aggravated PDGF-induced PCNA expression (a proliferation marker) and EdU incorporation while concomitantly inhibited the expression of cleaved-PARP and BAX/Bcl-2 (apoptosis markers), suggesting TXNRD1 plays a pivotal part in PDGF-BB-induced proliferation and apoptosis resistance in PASMC. Consistently, we then estimated whether TXNRD1 is essential for PDGF-BB-induced PASMC migration and the wound healing assay showed that TXNRD1 interference significantly accelerated the migratory quantity of PASMC (Figures 7F,G). In conclusion, these results suggest that TXNRD1 may protect PASMC from switching to a malignant phenotype following the PDGF-BB induction.

## Discussion

In this study, we performed an integrated bioinformatics analysis on four datasets containing 65 IPAH samples based on the RRA method. A total of 169 robust differential genes were screened, including 98 up-regulated genes and 71 down-regulated genes. GO and KEGG enrichment analysis showed that these DEGs were significantly enriched in some PAH-related functions and pathways. By constructing a PPI network and screening hub genes, we selected TXNRD1 for further research based on validation in independent datasets. We verified that the expression of TXNRD1 was decreased not only in the serum of IPAH patients but *in vivo* and *in vitro* experiments, which is in line with the predicted results. Furthermore, the GSEA analysis indicated that TXNRD1 may correlate with mTORC1 signaling pathway and MYC targets. Finally, *in vitro* experiments revealed that TXNRD1-knockdown PASMC exhibited exacerbated phenotype including hyperproliferation, migration and apoptosis resistance. This study is the first report to prove that TXNRD1 may be used as a clinical predictive molecule and potential therapeutic target for IPAH.

In recent years, more and more researchers have tried to explore the potential targets of pulmonary hypertension and explore the underlying pathogenesis through bioinformatics analysis. However, since some studies only adopted a single GEO dataset for analysis, or only take the intersection of the results, it may lead to bias and a large number of DEGs that have no biological effect. Regarding this issue, the RRA method has become a reliable method for obtaining important DEG (14). Based on the above integrated bioinformatics analysis, 10 significant hub genes have been identified. Among them, ENO1 and IDH1 were demonstrated to be aberrantly expressed in pulmonary hypertension, which provided strong support for our results. ENO1 encodes alpha-enolase, a glycolytic enzyme that catalyzes the conversion of 2-phosphoglycerate to phosphoenolpyruvate, which had been reported to mediate the malignant phenotypes of PASMCs in pulmonary hypertension, including hyperproliferation, apoptosis resistance, and metabolic conversion (15). Another document also confirmed that autoantibody against alpha enolase-1 in the blood of PAH patients with systemic lupus erythematosus could promote the proliferation and migration of PASMCs *in vitro* (16). IDH1 catalyzes the conversion of the citrate isomer isocitrate to alpha-ketoglutarate, which was reportedly elevated in PAH-PASMC (7). However, our results showed that ENO1 and IDH1 were reduced in IPAH patients, which was inconsistent with the above-mentioned literature. The possible reason is that the chip data measures the expression level at the transcription level rather than the protein level, and ENO1 and IDH1 may have post-transcriptional modification. Another possible reason is that the selected research objects are different: our research object is the lung tissue of IPAH patients, and the above-mentioned literature selected the isolated PASMCs from PAH patients for research.

In addition, it is worth noting that other hub genes including TALDO1, SHMT2, TKT, and PGD, are key enzymes involved in the pentose phosphate pathway (PPP), and their expression was inhibited in IPAH group. As we all know, aerobic glycolysis is one of the characteristics of PAH pathogenesis (17). Increased glycolysis can synthesize nucleotides *via* the pentose phosphate pathway to promote cell proliferation, thereby targeting the pentose phosphate pathway is one of the potential strategies to correct metabolic reprogramming. Similar to our results, Varghese et al. reported that the deficiency of the glucose-6-phosphate dehydrogenase (G6PD), the rate-limiting enzyme in PPP, could promote PAH development (18). Counterintuitively, G6PD deficiency did not reduce PPP flux but activated collateral pathways at the cost of increased oxidative stress. Combining the literature and our results, the inhibition of metabolic enzymes in PPP may be one of the unique molecular patterns of IPAH. However, apart from G6PD, genes related to PPP have not yet been elucidated in PAH. Therefore, the above-mentioned PPP-related genes that we have screened deserves to be further studied. These genes have been confirmed to regulate metabolic reprogramming in tumors (19, 20). Besides PPP-related genes, we also screened out several chemokines: CXCL10, CXCL9, CCL5. The dysregulation of chemokine and chemokine receptors had been shown to be implicated in PAH progression (21). Similar to our results, both CXCL10 and CXCL9 have been reported to have relatively elevated serum concentrations in IPAH patients (22, 23). While CCL5 is claimed to promote the PAH progression *via* the BMPR2 signaling pathway, and CCL5 receptor CCR5 has been reported to be involved in the interaction between macrophages and smooth muscle cells (24, 25).

Through the validation analysis of GSE113439, we identified TXNRD1 as the research object for further experimental verification. TXNRD1 is a critical antioxidant enzyme that catalyzes thioredoxin reduction to maintain the cell redox homeostasis (26). Most literatures reported that TXNRD1 was overexpressed in a variety of solid cancers (27, 28). The use of TXNRD1 inhibitors auranofin can overload tumor cells with ROS and promote tumor cell death (29). However, there were also literatures supporting that TXNRD1 could prevent tumorigenesis (30). At present, TXNRD1 has not yet been reported in pulmonary hypertension, while the substrate of TXNRD1, thioredoxin1 (Trx1), was claimed to be increased in hypoxia-induced PASMC proliferation (31), whereas Zimmer et al. observed that MCT-induced PAH animal model promoted a reduction in Trx1 (32). Potential reasons for this contradiction may arise from different stimuli or different stages of disease development. In addition, it is worth noted that numerous studies have reported the reduced expression or activity inhibition of antioxidant enzymes in PAH or PH animal model, such as SOD and GPXs (33). Our experimental results revealed for the first time that serum TXNRD1 of IPAH patients was lower than healthy controls and negatively correlated with mPAP and PVR, which suggested TXNRD1 could be a potential diagnostic marker for IPAH. Not only that, in the PAH animal model induced by MCT, the expression of TXNRD1 was also significantly decreased, which was in agreement with Zimmer’s observation. TXNRD1 inhibition had also been confirmed in PDGF-induced PASMC proliferation. Preliminary exploration of TXNRD1 function revealed that silencing of TXNRD1 exacerbated PDGF-induced uncontrolled proliferation, migration, and apoptosis resistance in PASMC. Since TXNRD is closely related to ROS production, we speculate based on our findings that the decreased expression of TXNRD1 increases the production of ROS to a certain extent, which can promote the proliferation of PASMC, but this hypothesis requires further research to prove.

In addition, through GSEA analysis of TXNRD1 related genes, we also found that TXNRD1 was closely correlated with mTORC1 signaling pathway, MYC targets and unfolded protein response, which provided some clues for future exploration of the mechanism involving TXNRD1 dysregulation in IPAH. mTORC1 is a serine/threonine protein kinase complex that regulates protein synthesis and acts as a cellular nutrient, energy, and redox sensor (34). It is reported that mTORC1 signaling is activated in PAH and required for PASMC proliferation induced by chronic hypoxia *in vivo* and *in vitro* (35, 36). mTORC1 activation can modulates cellular redox state through regulating SOD1 activity to ensure adequate proliferation while minimize oxidative damages (37). Myc is a well-known oncogenic transcription factor that serves as a downstream effector of many signaling pathways in PAH (38). Myc has been reported to potentially regulate the transcription of at least 15% of the entire genome (39), and the ChIP-seq data from the ENCODE consortium also demonstrated that Myc binds directly to the Nrf2 locus (an important antioxidant transcription factor) and increases its transcription (40). In view of this, we believe that the role of TXNRD1 in the pathogenesis of PAH be partly achieved by affecting the redox regulation mediated by mTORC1 or Myc. However, there is currently a lack of direct evidence to support this notion. At the same time, the correlation between TXNRD1 and mTOR as well as Myc has not yet been clarified in PAH, which deserves further research to validate.

There were some limitations in our study. First, the serum samples of IPAH patients we have collected are insufficient, so future studies consist of larger sample size are required to corroborate our results. Second, due to the limitations of laboratory conditions, the PAH animal model we used is relatively simple, so future researches are needed to verify the expression of TXNRD1 using other animal models such as hypoxia + Sugen5416 treated mice. Third, we did not conduct experiments to verify the activity of TXNRD1. Future studies are worth exploring the detailed mechanism of TXNRD1.

In conclusion, after integrating combined analysis by RRA method, we identified TXNRD1 as a potential biomarker for diagnosis of IPAH and potentially even a therapeutic target. Further research is needed to validate the function of TXNRD1 in IPAH pathogenesis.

## Data Availability Statement

The datasets presented in this study can be found in online repositories. The names of the repository/repositories and accession number(s) can be found in the article/Supplementary Material.

## Ethics Statement

The studies involving human participants were reviewed and approved by the Medical Ethics Committee of the Xiangya Hospital of Central South University. The patients/participants provided their written informed consent to participate in this study. The animal study was reviewed and approved by the Institutional Animal Care and Use Committee of Central South University.

## Author Contributions

ZY and LZ conceived and designed the study. WL performed this study and drafted the manuscript. YT and MZ reviewed the manuscript. BL and MW assisted in collecting clinical samples. All authors read and approved the manuscript.

## Conflict of Interest

The authors declare that the research was conducted in the absence of any commercial or financial relationships that could be construed as a potential conflict of interest.

## Publisher’s Note

All claims expressed in this article are solely those of the authors and do not necessarily represent those of their affiliated organizations, or those of the publisher, the editors and the reviewers. Any product that may be evaluated in this article, or claim that may be made by its manufacturer, is not guaranteed or endorsed by the publisher.

## Footnotes

1
https://www.ncbi.nlm.nih.gov/geo/

2
http://string-db.org

3
https://hiplot.com.cn/basic
