# Corrigendum: Integrated bioinformatic analysis reveals TXNRD1 as a novel biomarker and potential therapeutic target in idiopathic pulmonary arterial hypertension

**DOI:** 10.3389/fmed.2022.1116599

**Published:** 2022-12-15

**Authors:** Wenchao Lin, Yiyang Tang, Mengqiu Zhang, Benhui Liang, Meijuan Wang, Lihuang Zha, Zaixin Yu

**Affiliations:** Department of Cardiology, Xiangya Hospital, Central South University, Changsha, China

**Keywords:** bioinformatic analysis, TXNRD1, pulmonary hypertension, biomarker, RRA, GEO

In the published article, there was an error in the **Funding** statement “This study was supported by the National Natural Science Foundation of China (Nos. 8187011311, 81873416, and 8210012237) and the Key Research and Development Program of Hunan Province (2020SK2065).” The fund numbers (8187011311, 8210012237) were the initial acceptance numbers, not the final grant numbers. The correct **Funding** statement appears below.

