# Supplementary figures and images for: Integrated Bioinformatic Analysis Reveals TXNRD1 as a Novel Biomarker and Potential Therapeutic Target in Idiopathic Pulmonary Arterial Hypertension
Source: Front Med (Lausanne). 2022 May 12;9:894584. doi: 10.3389/fmed.2022.894584 (PMC9133447; doi:10.3389/fmed.2022.894584)

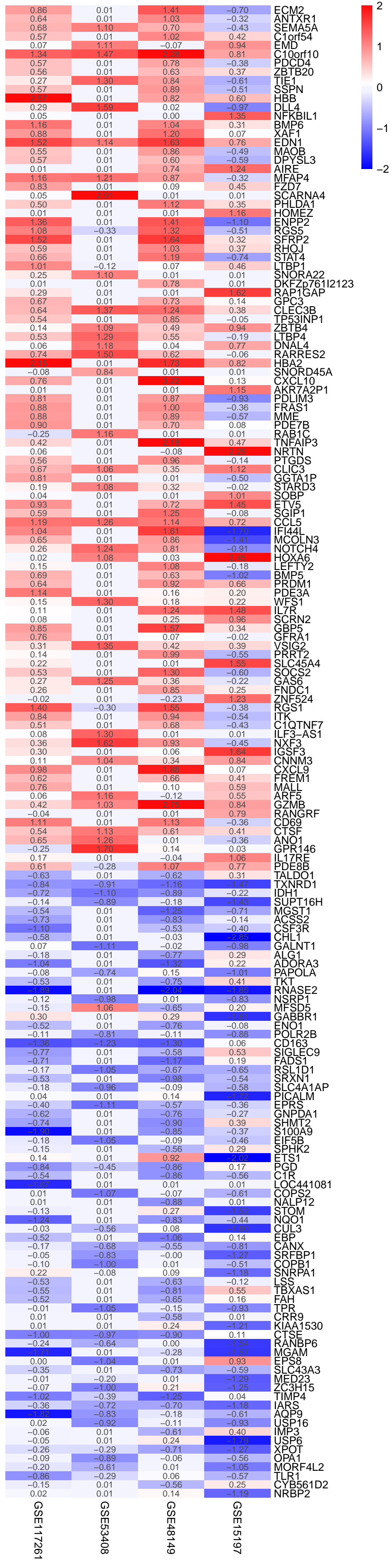

Supplement: Supplementary file 1 [file Data_Sheet_1.PDF]

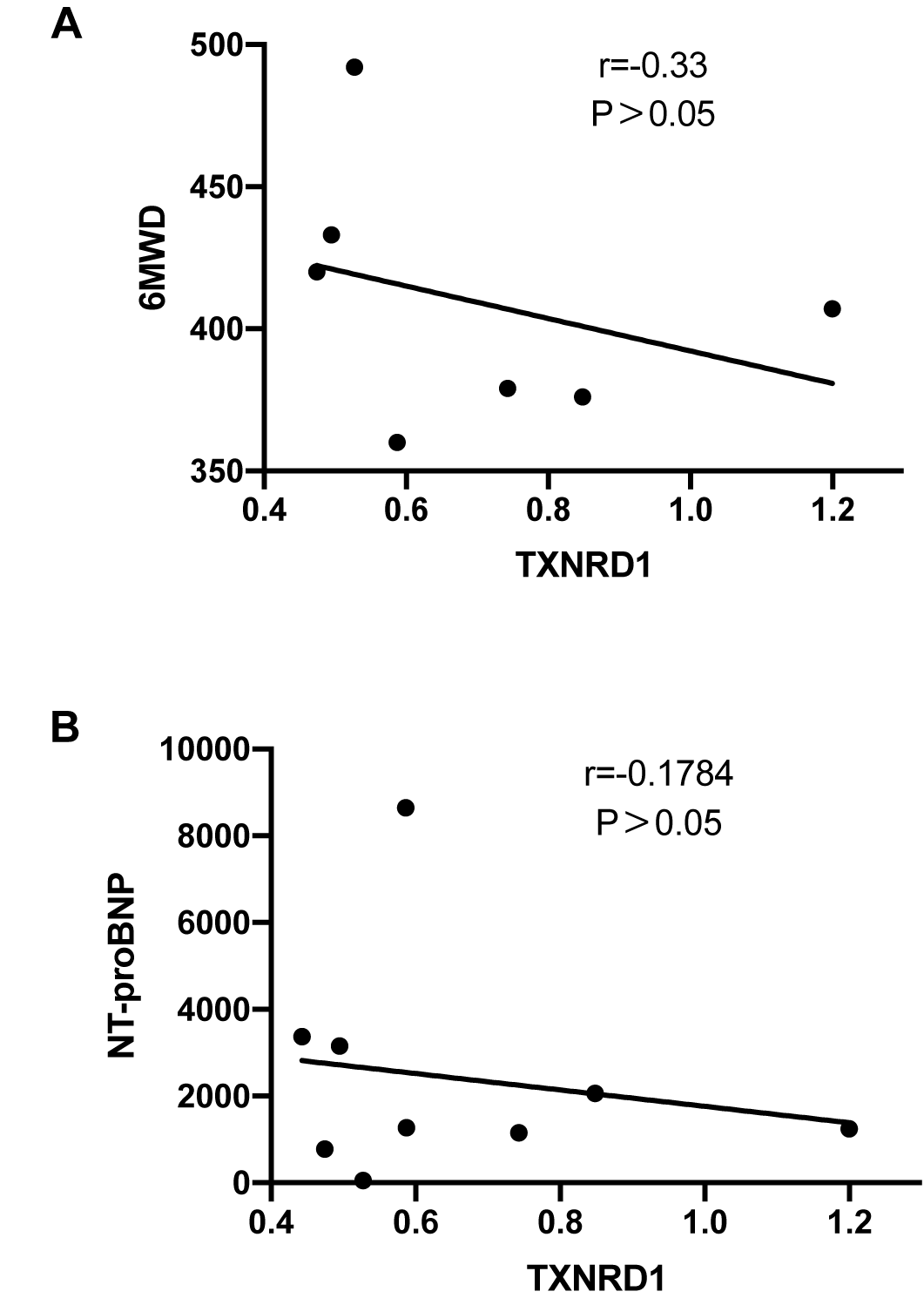

Supplement: Supplementary file 12 [file Image_1.TIF]

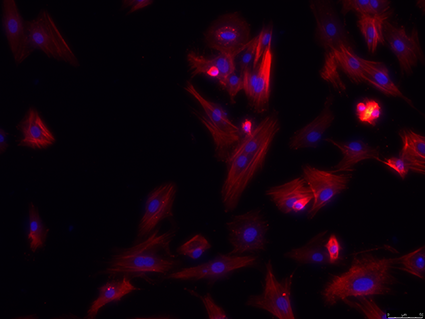

Supplement: Supplementary file 13 [file Image_2.TIF]
